# Denervation-induced dendritic reorganization leads to changes in the electrotonic architecture of model dentate granule cells

**DOI:** 10.1186/1471-2202-14-S1-P191

**Published:** 2013-07-08

**Authors:** Steffen Platschek, Hermann Cuntz, Mario Vuksic, Thomas Deller, Peter Jedlicka

**Affiliations:** 1 Institute of Clinical Neuroanatomy, Neuroscience Center, Goethe-University, Frankfurt/Main, D-60590, Germany; 2 Ernst Strüngmann Institute for Neuroscience in Cooperation with Max Planck Society, Frankfurt/Main, D-60528, Germany; 3 Croatian Institute for Brain Research, School of Medicine, University of Zagreb, Zagreb, HR-10000, Croatia

## 

Neuronal death and subsequent denervation of target areas is a major feature of several neurological conditions such as brain trauma, ischemia or neurodegeneration. The denervation-induced axonal loss results in reorganization of the dendritic tree of denervated neurons. Dendritic reorganization of denervated neurons has been previously studied using entorhinal cortex lesion (ECL).

ECL leads to shortening and loss of dendritic segments in the denervated outer molecular layer of the dentate gyrus [1]. However, the functional importance of these long-term dendritic alterations is not yet understood and their impact on neuronal electrical properties remains unclear. Therefore, in this study we analyzed what happens to the electrotonic structure and excitability of dentate granule cells after denervation-induced alterations of their dendritic morphology, assuming all other parameters remain equal.

To perform comparative electrotonic analysis we used computer simulations in anatomically and biophysically realistic compartmental models of 3D-reconstructed healthy and denervated granule cells. Our results show that somatofugal and somatopetal voltage attenuation due to passive cable properties was strongly reduced in denervated granule cells. In line with these predictions, the attenuation of simulated backpropagating action potentials and forward propagating EPSPs was significantly reduced in dendrites of denervated neurons. In addition, simulations of somatic and dendritic frequency-current (f-I) curves revealed increased excitability in deafferentated granule cells.

Taken together, our results indicate that unless counterbalanced by a compensatory adjustment of passive and/or active membrane properties, the plastic remodeling of dendrites following lesion of entorhinal cortex inputs to granule cells will boost their electrotonic compactness and excitability.
